# Relationship between Species Diversity and Community Stability in Degraded Alpine Meadows during Bare Patch Succession

**DOI:** 10.3390/plants12203582

**Published:** 2023-10-15

**Authors:** Yandi She, Xilai Li, Chengyi Li, Pengnian Yang, Zihan Song, Jing Zhang

**Affiliations:** College of Agriculture and Animal Husbandry, Qinghai University, Xining 810016, China; sheyandi0530@163.com (Y.S.); chengyi_li0801@163.com (C.L.); 18093559658@163.com (P.Y.); 18609718200@163.com (Z.S.); qhlxl2001@163.com (J.Z.)

**Keywords:** succession, patchily degraded alpine meadow, species diversity, community stability, stability evaluation

## Abstract

Plant diversity plays an important role in maintaining the stability of ecosystem functioning. Based on field surveys and indoor analyses, this study investigated the relationship between species diversity and community stability at different stages of bare patch succession in degraded alpine meadow ecosystems. Results show that: (1) Using the ICV (the Inverse of the Coefficient of Variation) method to analyze changes in plant community stability, community stability was generally ranked as follows: Long-term recovered patches > Healthy alpine meadow > Degraded alpine meadow > Short-term recovered patch > Bare Patches. (2) Using factor analysis to construct an evaluation system, the stability ranking based on species diversity was as follows: Healthy alpine meadow > Long-term recovered patches > Degraded alpine meadow > Short-term recovered patches > Bare Patches. (3) The community stability index was significantly positively correlated with vegetation coverage, height, biomass, species richness, Shannon–Wiener diversity index, species evenness, and Simpson’s diversity index (*p* < 0.05). Therefore, a positive correlation exists between plant diversity and community stability, such that plant communities with a higher species diversity tend to be more stable. To maintain the plant diversity and community stability of alpine meadow ecosystems, it is necessary to consider the characteristics of grassland plant composition and community structure, as well as their influencing factors, and promote the positive succession process of grasslands.

## 1. Introduction

As the main vegetation type in the Yellow River Source Zone, alpine meadows account for about 80% or more of the total area and play an important role as an ecological security barrier in water conservation, carbon sequestration and emission reduction, and biodiversity retention [1]. The degradation of alpine meadows in the Yellow River Source Zone, such as reduced vegetation coverage, loss of species diversity, and community stability, has been affected by climate warming and humidification and human activities in the past decades, and is spreading at a rate of 10.8% per year [2]. Patchily degraded alpine meadow is a critical stage to the formation of the black soil beach, in which the “hydrothermal hollow effect” leads to abnormal flow of the surface. Water and soil moisture and abnormal energy exchange with the atmosphere, which accelerates the alpine meadow degradation and causes significant differences in species diversity and community stability from the surrounding alpine meadows [3]. Therefore, studying the relationship between species diversity and community stability during bare patch succession in the Yellow River Source Zone provides theoretical guidance for the conservation and restoration of grasslands, and is important for maintaining ecosystem stability.

Species diversity is not only one of the important components of biodiversity, but also plays a great role in maintaining ecosystem functions, while plant community stability is one of the most basic functions of ecosystems, and the characteristics and change patterns of vegetation itself can be reflected by studying community stability [4,5]. Community stability and species diversity vary widely among successional stages, and the stability of a community directly responds to the current successional state of the community [6]. Species diversity and richness are the core of ensuring stability [7]. At present, there are three possible relationships between species diversity and stability: positive, negative, and correlated, which have not been uniformly understood. Wang et al. [8] and Campbell et al. [9] concluded that community stability and species diversity are positively and negatively correlated, respectively, while Giehl et al. [10] found no correlation between community stability and species diversity. In addition, the stability maintenance mechanism has been a hot topic in ecological research. Many important theories have been put forward, the most influential being the diversity–stability theory. It was first proposed by MacArthur and Elton, who found that the stability of ecosystems depends on the complexity and diversity of the system, and that large complex systems are more stable than simple systems. However, there are some doubts about the diversity–stability theory, as it suggests that a higher diversity affects the balance among species and leads to a decrease in stability [11]. Ouyang et al. [12] showed that the stability of grassland ecosystems is largely influenced by species diversity and that degradation of grassland ecosystems reduces their ability to maintain system stability. Therefore, it is uncertain whether the reduction in species diversity at the alpine meadow patchy successional stages in the Yellow River Source Zone caused by degradation will lead to a reduction in the stability of the responding communities.

Community succession is a dynamic process, in which some species replace others, and one species community replaces another over time [13]. Community stability is a dimension of ecosystem stability, which can be defined as the ability of a community to resist external disturbances when disturbed and the ability of a system to maintain its original structure or state after a disturbance, including status quo stability, time course stability, resilience stability, etc. The mechanisms regarding the maintenance of stability are complex due to conceptual differences and different approaches [14]. Usually, community stability is related to self-replication, and communities that tend to top succession tend to have higher community stability, but the succession process may also be influenced by a combination of intra- and interspecific variations and environmental or anthropogenic disturbances, among other factors [15]. Therefore, we assumed that species diversity is positively correlated with community stability during patchy succession in this study. Five stages of alpine meadow patch succession were studied to explore the correlation between species diversity and stability, and to evaluate community stability based on species diversity, so as to provide references for vegetation restoration and ecological construction of degraded alpine meadows in the Yellow River Source Zone.

## 2. Materials and Methods

### 2.1. Study Area

**Figure 1:**
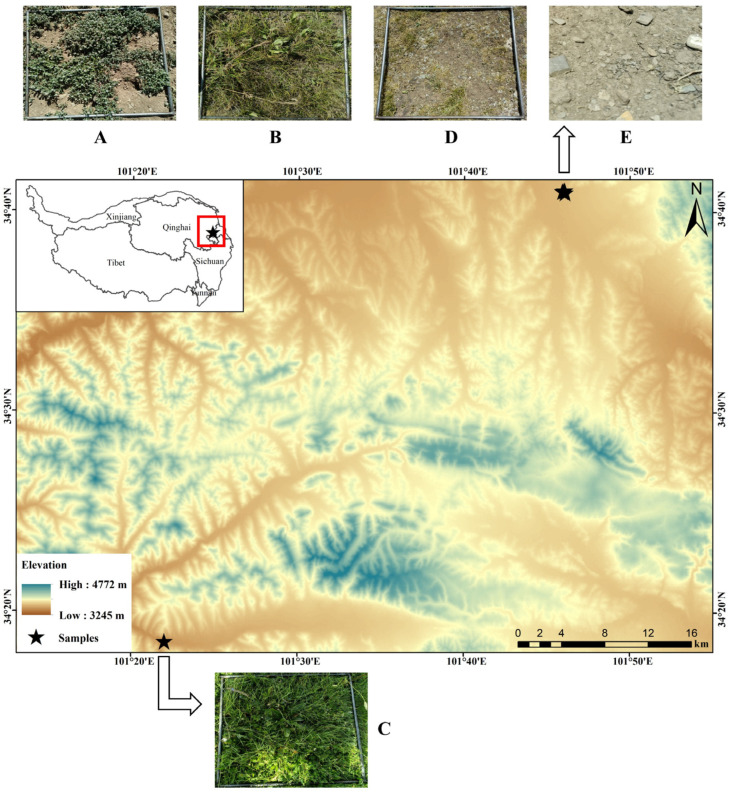
Bare patch succession stages of alpine meadow ((**A**) Short-term recovery patch; (**B**) Long-term recovery patch; (**C**) Healthy alpine meadow; (**D**) Degraded alpine meadow; (**E**) Bare Patches).

The test site is located in Henan Mongolian Autonomous County, Huangnan Tibetan Autonomous Prefecture, Qinghai Province, China, with the geographical coordinates of 34°05′52″–34°56′36″ N and 100°53′06″–102°16′12″ E. The elevation of the county is 3600–4200 m. The topography is high in the northeast and low in the southwest, mainly including four types of landforms: beaches, hills, low mountains, and high mountains. The climate is a continental plateau climate, with a warm and rainy season from May to October and a cold, dry, and windy season from November to April. The average annual temperature ranges from −1.3 to 1.6 °C, and the annual precipitation ranges from 597.1~615.5 mm. The main vegetation type in this area is alpine meadows dominated by *Kobresia*, including *Kobresia humilis*, *Kobresia capillifoliae*, and *Kobresia pygmaea*. The soil type is mainly alpine meadow soil with a rich content of organic matter, and the potential fertility of soil is high, but the activity of soil microorganisms is weak, the rate of nutrient transfer is low, and the turnover time is long because of the hypoxia, poor efficiency of fertilizer supply. A large number of plateau pika (*Ochotona curzoniae*) and plateau zokor (*Eospalax baileyi*) are distributed in the study area. The plateau pika and plateau zokor foraging and burrowing have created a large number of bare patches in the alpine meadow in this area (Figure 1).

### 2.2. Methods

#### 2.2.1. Sample Plot Setup

In this study, five successional stages of short-term recovery patches, long-term recovery patches, healthy alpine meadows, patch degraded alpine meadows, and bare patches were selected randomly in the sampling area in August 2022, and the patches were classified according to vegetation type and total vegetation coverage (Table 1). Short-term recovery patches are patches formed after the plateau sage-grouse stopped disturbing activities for 1~2 a, with a few first- and second-year miscellaneous grasses, such as *Elsholtzia densa* and *Ajania tenuifolia*, with vegetation coverage of 20~40%; long-term recovery patches are patches formed after short-term recovery patches. Long-term recovery patches are patches formed via further natural ecological recovery, mainly dominated by *Poa annua* and *Carex atrofusca* and *Kobresia humilis*, accompanied by a large number of perennial forbs, such as *Anaphalis lactea*, with a vegetation coverage >40%; The vegetation coverage of healthy meadow is >90%, and the vegetation coverage of degraded alpine meadow is dominated by *Cyperaceae* of *Carex atrofusca* and *Kobresia humilis*, accompanied by a large number of *Anaphalis lactea*, and the vegetation coverage is 40~60%. A standard sample plot with an area of 50 cm × 50 cm (herbaceous) was set up to survey the plant community in the field and record the names and numbers of the species occurring therein, with reference to Flora Reipublicae Popularis Sinicae (http://www.iplant.cn/, accessed on 20 May 2023), Flora of Qinghai and Flora of Henan-Mongolian Autonomous County, Chinese Virtual Herbarium, and The Plant List (http://www.theplantlist.org/, accessed on 25 May 2023) to double-check the Latin names of the species (to avoid errors caused by factors such as adjustment of families and multiple species).

#### 2.2.2. Vegetation Survey and Sampling

**Figure 2:**
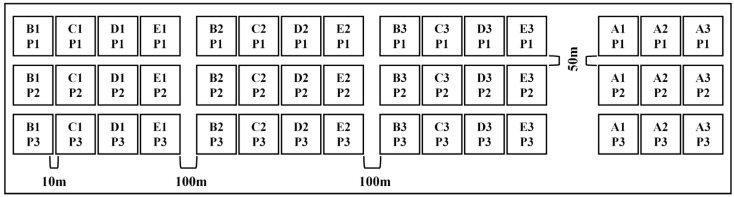
Distribution of sampling plots and the combination of different treatments.

Three random 50 cm × 50 cm sample squares were set in each sample plot for vegetation survey, and to avoid the edge effect, the interval between adjacent sample squares was set at >10 m (Figure 2). Three replicates were spatially set for each successional stage, totaling nine replicates. All plants in the sample plots were identified, and the coverage of each species and the total coverage of the sample plots were visually estimated, and the height of each species was measured by straightedge and recorded.

#### 2.2.3. Species Diversity Index

In community analysis, the importance value (*IV*) can be used as a measure of plant species dominance. The relative coverage (*RC*), relative height (*RH*), and relative abundance (*RA*) of individual species were used to calculate the important values of each species:

*RC* (%) = The coverage of a species/coverage of all species
*RH* (%) = The height of the individual of a species/the sum of the individual heights of all species
*RA* (%) = The specie of the number of individuals/number of individuals of all species

(1) $$IV=\left(RC+RH+RA\right)/3,$$

The Patrick richness index, Shannon–Wiener diversity index, Pielou evenness index, and Simpson dominance index were used to calculate the species diversity:

Patrick richness index (*R*)

(2) R=S,

Shannon–Wiener diversity index (*H*)

(3) $$H=\sum_{i=1}^{S}\left(P_{i}\ln P_{i}\right),$$

Pielou evenness index (*E*)

(4) $$E=\frac{H}{\ln S\;},$$

Simpson dominance index (*D*)

(5) $$P_{i}=\frac{{IV}_{i}}{{IV}_{total}},$$

(6) $$D=1-\sum_{i=1}^{S}P_{i}^{2},$$

where *s* is the sum of all the plant species in the sample plot (or square); *I* is the plant species in the sample square; and *P_i_* is the relative importance of the ith species.

#### 2.2.4. Plant Community Stability

Plant community stability is expressed as the inverse of the coefficient of variation (CV) of the relative coverage of the community species (*ICV*) [16],

(7) ICV=μ/σ,

In the formula, *μ* is the relative coverage of each species in the sample square, and σ is the standard deviation of the relative coverage of each species. The lower the value of *ICV*, the lower the stability of the plant community, and conversely, the higher the stability of the plant community. Stability Assessment uses factor analysis, a method for studying the internal dependencies of the correlation matrix between multiple variables to identify several random factors that combine key information about all variables and represent the underlying structure of the data. The factors being measured are uncorrelated with each other, and all variables can be represented as a linear combination of common factors. Taking n samples, *p* indicators, and *X* = (*X*_1_, *X*_2_…, *X*_P_) as the random variables, the model is expressed as follows.

(8) $$\begin{array}{c} X_{1}=a_{11}F_{1}+a_{12}F_{2}+\cdots +a_{1m}F_{m}+\mu_{1},\;\\ X_{2}=a_{21}F_{1}+a_{22}F_{2}+\cdots +a_{2m}F_{m}+\mu_{2},\\ \;\cdots ,\;\;\\ X_{p}=a_{p1}F_{1}+a_{p2}F_{2}+\cdots +a_{pm}F_{m}+\mu_{p} \end{array}$$

where matrix A (aij) is called the factor loading matrix, aij is the factor loading, and μ is the special factor representing the variance of the variables that cannot be explained by the common factors and can be ignored in the actual analysis. In the operation process, factor analysis was performed on the variable dataset using econometric statistical analysis software, and the combined scores of the factors were finally determined by testing Bartlett’s spherical and Kaiser–Keyer–Olkin statistics of the samples and measuring the main factors using the maximum variance method of rotation.

Microsoft Excel 2010 was used to collate and calculate the data. SPSS 26.0 was used to carry out one-way statistical analysis of variance and correlation analysis. Duncan’s multiple comparison test was used, the Pearson correlation analysis method was used for correlation analysis, *p* < 0.05 indicated that the difference was significant, *p* < 0.01 indicated that the difference was extremely significant, and Origin 2023 software was used for producing graphics.

## 3. Results

### 3.1. Diversity of Plant Composition

#### 3.1.1. Important Value

A total of 50 herbaceous plant species was found in the process of bare patch succession in the alpine meadows (Table 2). Only *Elsholtzia densa* was found to have an important value of more than 0.2 during the short-term recovery of bare patch succession in the alpine meadows; the important value was 0.36. In the long-term recovery patches, the important value was 0.21, 0.24, and 0.30 for *Elymus nutans*, *Poa annua*, and *Koeleria macrantha*, respectively. In healthy alpine meadows, *Elymus nutans* and *Potentilla chinensis’s* importance values were 0.23 and 0.25, respectively. *Kobresia humips*, with an important value of 0.39, was found to be more than 0.2 in the degraded alpine meadow. No plants were found in bare patches. With succession, the plants in the community changed from annual forbs to Gramineae + perennial forbs, Gramineae + Cyperaceae + forbs, and Cyperaceae + forbs to no-plants.

#### 3.1.2. Functional Group Structure of Plant Communities

**Figure 3:**
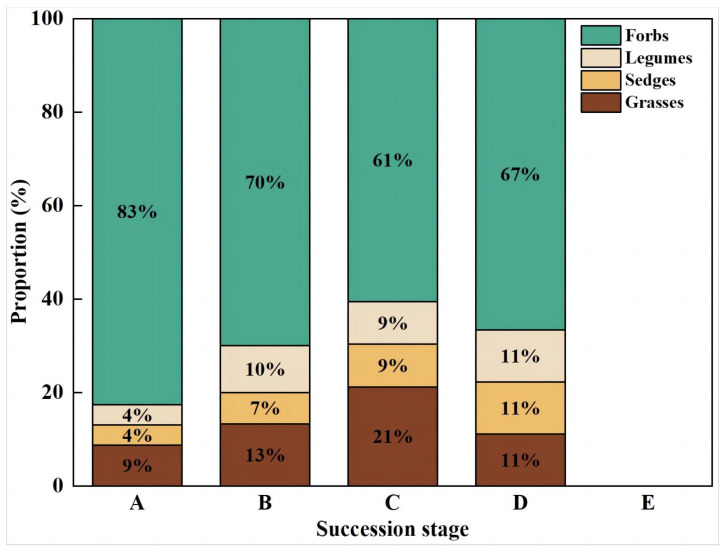
Proportion of functional group changes at four bare patch succession stages of the degraded alpine meadow ((**A**) Short-term recovery patch; (**B**) Long-term recovery patch; (**C**) Healthy alpine meadow; (**D**) Degraded alpine meadow; (**E**) Bare Patch).

The composition of the plant communities at different successional stages (Figure 3) is described as Gramineae, accounting for C (21%) > B (13%) > D (11%) > A (9%) > E (0%), and sedge for D (11%) > C (9%) > B (7%) > A (4%) > E (0%). The proportion of Legumious was D (11%) > B (10%) > C (9%) > A (4%) > E (0%). The proportion of frobs followed the order of A, (83%) > B (70%) > D (67%) > C (61%) > E (0%). In contrast to short-term recovery patches, long-term recovery patch-healthy meadows, Gramineae increased, whereas forbs decreased, healthy meadows-degraded-bare patch, Gramineae decreased, and forbs increased. Cyperaceae and Leguminous plants increased during the whole succession stage and reached the highest level after the degradation of healthy alpine meadow.

#### 3.1.3. Changes in Plant Community Coverage and Biomass

**Figure 4:**
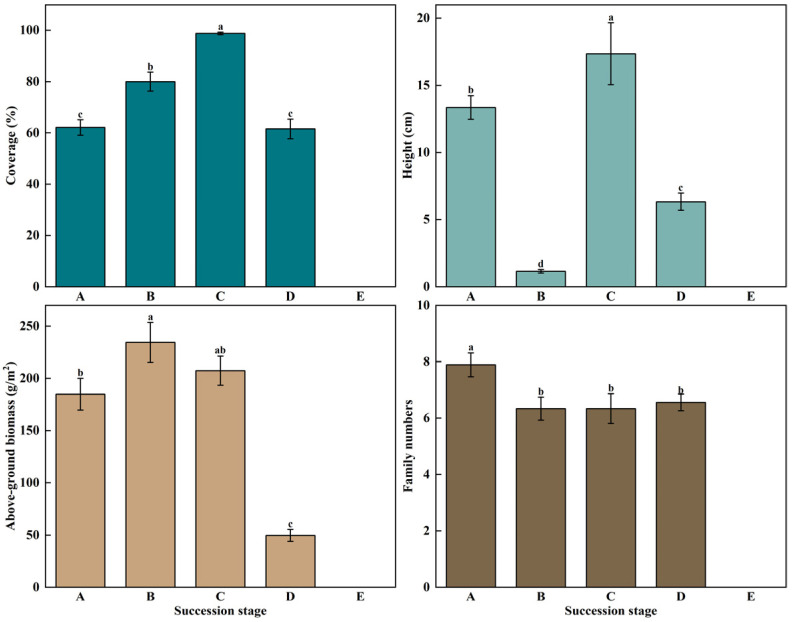
Changes of plant coverage, height, biomass, and family number in the degraded alpine meadow during bare patch succession ((**A**) Short-term recovery patch; (**B**) Long-term recovery patch; (**C**) Healthy alpine meadow; (**D**) Degraded alpine meadow; (**E**) Bare Patch), different lowercase letters represent significant differences in plant community coverage and biomass in the degraded alpine meadow during bare patch succession (*p* < 0.05).

During the succession, the coverage of the vegetation community increased significantly from short-term recovery to healthy meadow (*p* < 0.05), because of the influence of climate change and human activities. The healthy meadow gradually degenerated to become bare land after a long succession (coverage is 0), during which the coverage of the vegetation community showed a significant decrease trend (*p* < 0.05). During succession, the height of the vegetation community showed a significant changing trend (*p* < 0.05), and the plant height at the short-term recovery stage dominated by annual forbs was higher than that of plants at the long-term recovery stage dominated by perennial forbs and Gramineae (*p* < 0.05). The vegetation height of healthy meadow composed of Gramineae + Cyperaceae + forbs was higher than that of degraded meadow composed mainly of Cyperaceae + forbs, and Gramineae decreased (*p* < 0.05). In the process of succession, the aboveground biomass at the long-term recovery stage was significantly higher than that at other succession stages (*p* < 0.05), which followed the order of B > C > A > D > E. During the succession process, the number of families at the short-term recovery stage was significantly higher than that at other succession stages, but there was no significant difference among long-term recovery stage, healthy meadow, and degraded meadow (Figure 4).

### 3.2. Plant Species Diversity during Vegetation Succession

**Figure 5:**
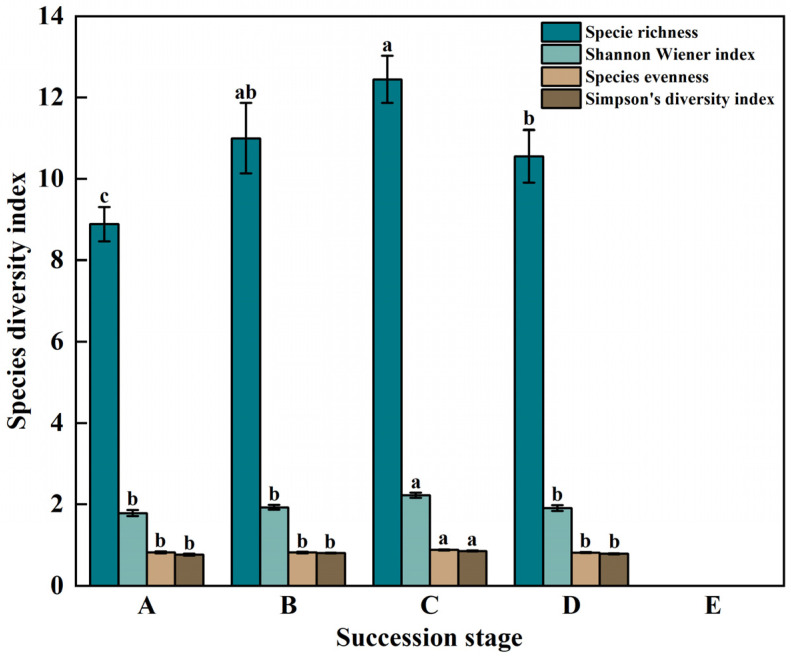
Changes of Species Diversity Index in bare patch succession in the degraded alpine meadow ((**A**) Short-term recovery patch; (**B**) Long-term recovery patch; (**C**) Healthy alpine meadow; (**D**) Degraded alpine meadow; (**E**) Bare Patch), different lowercase letters represent significant differences in plant species diversity in the degraded alpine meadow during bare patch succession (*p* < 0.05).

During succession, the species richness index ranged from 0.00 to 12.44, and the difference at different successional stages was significant (*p* < 0.05). The species richness of healthy meadow was the highest; there is no species in bare patches. The Shannon–Wiener Diversity Index ranged from 0.00 to 2.22, and there were significant differences between the healthy meadow stage and other stages (*p* < 0.05), but there was no significant difference among other stages (*p* > 0.05). The evenness index of all stages was between 0.00 and 0.88, and the difference between the healthy meadow stage and other stages was significant (*p* < 0.05), but there was no significant difference among other stages (*p* > 0.05). The Simpson diversity index lay between 0.00 and 0.86 at all stages, and there were significant differences between the healthy meadow stage and other stages (*p* < 0.05), but not among other stages (*p* > 0.05) (Figure 5).

### 3.3. Analysis of Plant Community Stability

**Figure 6:**
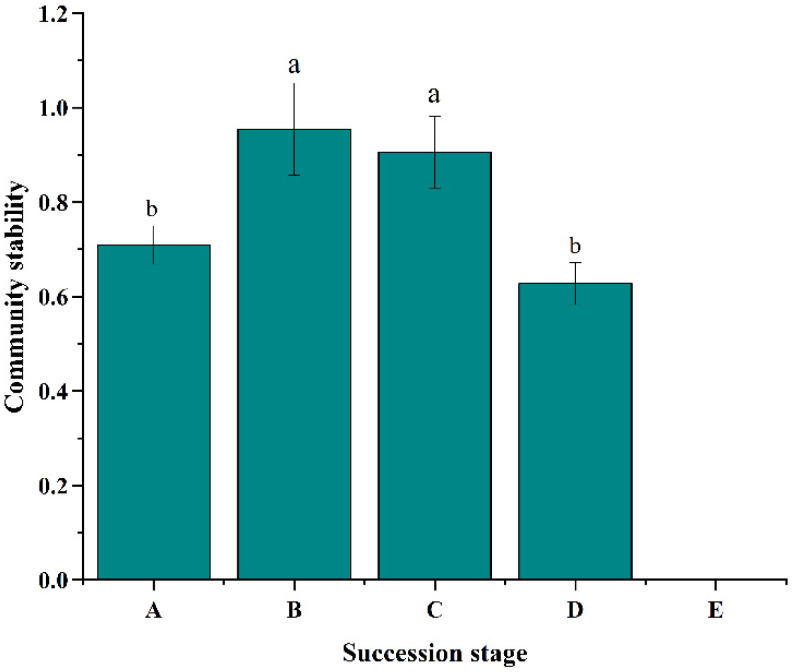
Changes in the community stability index during bare patch succession in the degraded alpine meadow ((**A**) Short-term recovery patches; (**B**) Long-term recovery patches; (**C**) Healthy alpine meadow; (**D**) Degraded alpine meadow; (**E**) Bare patches), different lowercase letters represent significant differences in plant community stability in the degraded alpine meadow during bare patch succession (*p* < 0.05).

As the level of community stability increases, the community’s ability to resist the invasion process strengthens, thereby reducing the invasiveness of weeds. The results showed that there were significant differences in community stability indices at different succession stages of bare patches in the degraded alpine meadow (Figure 6); the community stability followed the order of long-term recovery patches > healthy meadow > degraded alpine meadow > short-term recovery patches > bare patches.

### 3.4. Relationship between Plant Species Diversity and Community Stability

**Figure 7:**
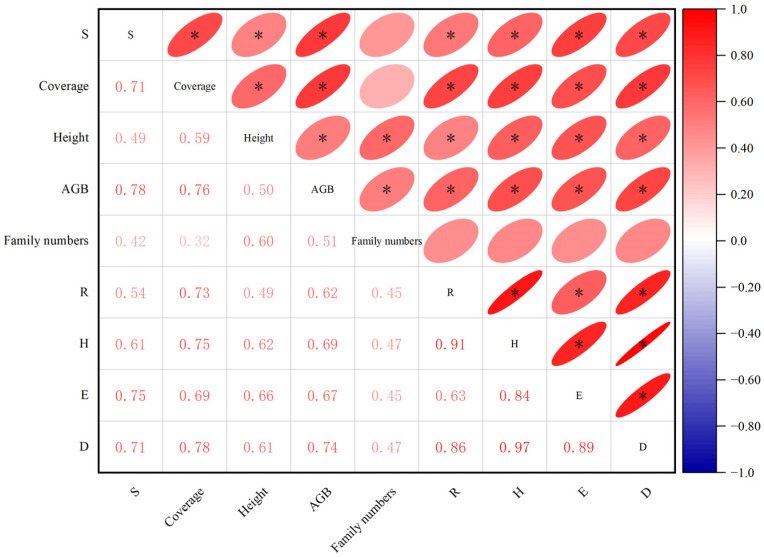
Correlation between community stability and other characteristics at various bare patch succession stages of the degraded alpine meadow (S—community stability; R—species richness; H—Shannon–Wiener Diversity Index; E—species evenness; D—Simpson Diversity Index), * represents significant correlation at the level of α = 0.05.

Shown in Figure 7 are the results of correlation analysis between community stability and coverage, height, biomass, family numbers, and species diversity. It can be seen that the community stability index was positively correlated with coverage, height, biomass, species richness, Shannon–Wiener Diversity Index, species evenness, and Simpson diversity index (*p* < 0.05). Among these, community stability had the highest correlation coefficients with cover, biomass, E, and D. This is due to the fact that communities with high species diversity are more likely to contain species with asynchronous dynamics and more stable productivity. This was followed by R and height, while correlation coefficients with Family numbers were the lowest and non-significantly positive, probably due to the insignificant effect on community stability at the plant family level.

### 3.5. Evaluation of Community Stability under Bare Patch Succession

Factor analysis was used to evaluate the community stability at the five stages of patch succession in the degraded alpine meadow, and four indexes were included in the evaluation. They are species richness, Shannon–Wiener Diversity Index, species evenness, and Simpson Diversity Index. Before factor analysis can be performed, the raw data need to be standardized to eliminate the effect of dimension on the final factor analysis results, followed by validation of data applicability by Kaiser–Meyer–Olkin (KMO) and Bartlett spherical tests. The test results are shown in Table 3; the KMO value is 0.602, and the Chi-square statistical significance probability of the Bartlett spherical test is 0.000. The results showed that the correlation of each index was good and suitable for factor analysis.

The explanation (%) of the total variance is shown in Table 4. It can be seen from the sum of the squares of the rotating loads and the loading that the two principal component factors are extracted according to the eigenvalues greater than 1, and their variance contribution rates are 53.81% and 46.07%, respectively. The cumulative contribution rate is 99.88%, which indicates that the two principal components contain 99.88% of the information of the original data. Therefore, it is suitable to use these two principal components to evaluate the community stability of patch succession in the degraded alpine meadow.

The component matrix after rotation is shown in Table 5. For the first principal factor, the species Evenness Index, the Simpson Diversity Index, and the Shannon–Wiener Diversity Index all have high loading values, which are 0.844, 0.794, and 0.714, respectively. The species richness index of the second factor *F*2 was 0.836, and the higher the loading value, the better the explanatory power.

The factor score coefficient matrix is shown in Table 6. The main function of the matrix is to score each common factor:

(9) F1=−1.463X1−0.014X2+1.390X3+0.805X4, F2=1.955X1+0.394X2−1.136X3−0.496X4 

The weight of the two common factors is calculated according to the variance decomposition table (Table 7). That is, the variance contribution rate of the common factors divided by the sum of the variance contribution of the two common factors equals ω1 = 0.539 and ω2 = 0.461, respectively; the comprehensive formula is f = 0.789 *F*1 + 0.210 *F*2.

Based on the factor analysis of the Community Stability Evaluation Index system, the results showed that the Shannon–Wiener Diversity Index, species evenness index, and Simpson Diversity Index had the greatest influence on the community stability; secondly, the species richness index also had some influence on the community stability. The results showed that the community stability had the following order of healthy meadow > long-term recovery patches > degraded alpine meadow > short-term recovery patches > bare patches.

## 4. Discussion

### 4.1. Changing Plant Community Characteristics of Bare Patch Succession

With global climate change and increased population, human activities have seriously disrupted the balance of the ecosystem, affected biodiversity, and led to the fragmentation of species habitats. In turn, habitat fragmentation leads to the decline of biodiversity and vegetation distribution in patches [17,18,19]. There are a number of experimental studies showing that communities with high species richness have a higher community productivity and are more resistant to invasive species and have more stable ecosystem functions [20,21]. The bare patch succession in grassland is not homogeneous, but a grassland ecosystem with a mosaic distribution of recovered patches at various succession stages. The vegetation community characteristics of bare patches at different recovery stages are obviously different [22,23]. In this study, coverage, height, biomass, and family number changed with the succession of bare patches in the alpine meadow. At the long-term recovery stage, perennial forbs were dominant because the community was relatively stable, and the perennial plants have a strong ability of clonal reproduction and ecological adaptability, through clonal growth, effectively occupying a larger space. The early recovery stage belongs to the early stage of succession, and the competition of different plant species for natural resources to survive is intense. With the succession, the species in the community may have intense competition [24]. In order to reduce competition, species have some separation between them, so as to make the community stable. Most species in a community compete for light, heat, water, and nutrients. With the depletion of resources, competition will intensify, resulting in the loss of plant diversity [25].

Plant species diversity is the basis of maintaining the stability and production of the grassland ecosystem, which will lead to the change in the structure and functions of the grassland ecosystem during the succession of bare patches in the degraded alpine meadow [26,27]. The species diversity index is usually used to comprehensively reflect the equilibrium of species richness and population in a community. Most studies have shown that in the process of succession from bare or abandoned land to native community, the diversity index increased gradually, but decreased when the community was near the top [28]. The patchily degraded alpine meadow is at a kind of mid-succession stage, and the main species of each stage have not changed obviously, but the whole succession mainly displays the change of species in each original component community. In the succession process, the species are in a dynamic development process, the balance between the vegetation layer or species has not been established, and the whole community structure is not stable, so the stability of the community is weak [3,29]. Therefore, species diversity changed significantly during the whole succession, except for species richness. The Shannon–Wiener Diversity Index, species evenness index, and Simpson Diversity Index did not change in bare patches, and short-term and long-term recovery patches.

### 4.2. Relationship between Community Stability and Other Characteristics

The stability of a community is regulated by competition and symbiosis among different species in the community, which eventually leads to the concentration of dynamic equilibrium. Plant community stability is mainly affected by interspecific competition, environmental pressure, and disturbance activities [30]. In this study, community stability was significantly correlated with the coverage, height, and biomass of vegetation. Based on coverage, community stability was calculated. Coverage and height were the decisive factors of vegetation aboveground biomass. With the increase in plant coverage, the thermal radiation of the soil surface is slowed down, the evaporation of water on the surface is reduced, the illumination of the vegetation on the surface is reduced, the survival rate of seedlings and seeds is changed, and the species composition of the community is changed, thereby improving community stability [31].

Species richness reflects the diversity of species in a community, the Shannon–Wiener Diversity Index and Simpson Diversity Index reflect the structure of a community; the species evenness index reflects the evenness of individual species distribution and composition in a habitat or community. The relationship between species diversity and stability is controversial. MacArthur and Elton developed the diversity–stability hypothesis, which holds that species diversity and community stability have a positive effect—the more resistant to external disturbance, the better for community and ecosystem stability [32,33]. Species diversity can contribute to the stability of ecosystem functions [34], and species diversity is the key to the ecosystem stability [35]. In this study, community stability was significantly correlated with species richness, Shannon–Wiener Diversity Index, species evenness, and Simpson Diversity Index. Species diversity includes two aspects of species richness or the number of species in a given area, as well as species evenness or the distribution of species biomass in a community [36]. The stability of the plant community depends on the resistance and self-repairing ability of the plant community and its internal structure and species functions with regards to disturbance. To a great extent, the ecological compensation effect between functional groups is also obvious, thus maintaining grassland community stability effectively [37]. Because the calculated stability is based on all the species and species coverage of the community, and the stability is disturbed by the external environment, so the key to maintain the stability of the community is to reduce the disturbance of the external environment. The measure of stability or the relationship between diversity and stability cannot be explained by a single factor, and the change of stability is more likely to be caused by the joint influence of diversity and other factors.

### 4.3. Evaluation of Community Stability

The species diversity of the grassland vegetation community reflects the stability and complexity of the community, and the stability of the community can be evaluated simply by constructing the evaluation system of species diversity of the grassland community [38]. However, community succession is a long-term and complex ecological process of interactions between the community and the external environment. The stability of the community is determined by many factors within the community. According to Clements’ succession theory, community succession in any area must be directed to the top level and eventually to the top level after a long succession; an undisturbed grassland community develops from a stage with a simple, unstable, or less stable structure to a stage with a more complex and stable structure [39]. The results of this study were as follows: short-term recovery from bare patch-annual forbs-long-term recovery from perennial forbs-near-undegraded healthy meadow composed of Gramineae +Cyperaceae +forbs-patch degradation from Cyperaceae. With the succession, the composition of the plant community changed greatly, and the succession of dominant species reflected the changing characteristics of the community ecosystem structure and functions in the process of vegetation recovery; the recovered community gradually evolved to a community type of healthy alpine meadow, indicating a more stable community development direction. As a whole, with the succession, the dominant species succession is obvious, the community succession takes place obviously, and the species diversity and the community stability present the positive correlation. The community succession is a long-term and dynamic process [40,41]. In this study, we used the method of space instead of time to analyze and discuss succession. The evaluation of plant community composition and stability at the bare patch succession stage of the alpine meadow is only a preliminary result, and the characteristics of plant community composition and the stability of bare patch succession in the alpine meadow need to be further studied.

## 5. Conclusions

In the process of bare patch succession of the degraded alpine meadow, there is a positive correlation between species diversity and community stability. With the development of recovery succession, the species and structure of the plant community become more abundant and more complex, and the stability of the plant community becomes stronger. In addition, according to the evaluation results, the composition of the plant community changes greatly with the progress of succession, and the succession of dominant species reflects the changing characteristics of the structure and functions of the community ecosystem in the process of bare patch succession of the degraded alpine meadow. It indicated that the community developed in a more stable direction. The results show that the species diversity and stability of bare patch succession in the degraded alpine meadow are mutually restricted and synergistic during the natural recovery. Therefore, in the context of future global change and biodiversity loss, studying the impacts and mechanisms of biodiversity on ecosystem stability at both the temporal and spatial scales is more conducive to understanding and recognizing the relationship between biodiversity and ecosystem functioning and stability at the landscape scale.

## Figures and Tables

**Table 1:** Basic information of sample sites.

Treatment	Successional Stage	Longitude, Latitude	Altitude/m
A	Short-term recovery patch	34°41′4″, 101°46′3″	3609
B	Long-term recovery patch	34°41′2″, 101°45′55″	3606
C	Healthy alpine meadow	34°18′30″, 101°22′0″	3494
D	Degraded alpine meadow	34°40′58″, 101°46′4″	3610
E	Bare patch	34°41′3″, 101°46′0″	3604

**Table 2:** Important values of plant species in the process of patch succession of alpine meadow.

Family	Species	Importance Values				
		A	B	C	D	E
Gramineae	*Elymus nutans*	0.17	0.21	0.23	0.11	—
	*Deschampsia cespitosa*	—	0.07	0.07	—	—
	*Stipa capillata*	—	—	0.05	—	—
	*Poa annua*	0.14	0.24	0.16	0.09	—
	*Agrostis canina*	—	—	0.07	—	—
	*Leymus chinensis*	—	—	0.11	—	—
	*Koeleria macrantha*	—	0.30	0.12	—	—
Cyperaceae	*Carex* spp.	0.12	0.13	0.10	0.07	—
	*Kobresia humips*	—	0.16	0.07	0.39	—
	*Kobresia humilis*	—	—	0.06	—	—
Composite	*Taraxacum mongolicum*	0.04	0.02	0.03	0.02	—
	*Cirsium arvense var. integrifolium*	0.06	0.03	—	—	—
	*Ajania tenuifolia*	0.10	0.03	0.02	0.03	—
	*Leontopodium leontopodioides*	—	0.07	0.03	0.11	—
	*Saussurea superba*	0.02	0.08	0.07	0.08	—
	*Pedicularis resupinata*	0.09	0.03	—	—	—
	*Ligularia virgaurea*	0.02	0.05	—	0.11	—
	*Aster tataricus*	—	—	0.08	—	—
	*Anaphalis lactea*	—	0.06	—	0.11	—
Rosaceae	*Potentilla anserina*	—	0.08	0.07	—	—
	*Parnassia palustris*	—	—	0.03	—	—
	*Potentilla chinensis*	—	0.04	0.25	0.04	—
	*Potentilla discolor*	—	0.02	0.05	0.06	—
	*Potentilla pamiroalaica*	—	—	0.06	—	—
Leguminous	*Oxytropis tianschanica*	—	0.01	0.05	0.05	—
	*Tibetia himalaica*	—	0.01	0.03	—	—
	*Astragalus membranaceus*	0.03	0.04	—	0.06	—
	*Medicago Sativa*	—	—	0.07	—	—
Ranunculaceae	*Ranunculus japonicus*	0.04	0.02	0.06	—	—
	*Delphinium caeruleum*	—	—	0.05	—	—
	*Aconitum carmichaelii*	0.06	—	—	—	—
Lamiaceae	*Elsholtzia densa*	0.36	—	—	—	—
	*Ajuga lupulina*	0.09	0.04	—	—	—
Gentianaceae	*Gentiana straminea*	—	0.04	0.03	0.09	—
	*Halenia corniculata*	0.04	0.04	0.07	0.05	—
	*Gentiana scabra*	—	0.03	—	0.05	—
Scrophulariaceae	*Veronica polita*	0.07	0.03	—	—	—
	*Lancea tibetica*	0.10	0.06	0.01	0.04	—
	*Lagotis brachystachy*	—	—	0.02	—	—
Umbelliferae	*Pleurospermum uralense*	—	—	0.05	—	—
	*Daucus carota*	0.03	0.02	—	—	—
Plantaginaceae	*Plantago asiatica*	—	0.02	0.03	—	—
Caryophyllaceae	*Stellaria media*	—	0.01	—	—	—
Brassicaceae	*Descurainia sophia*	0.08	—	—	—	—
Thymelaeaceae	*Stellera chamaejasme*	—	—	0.05	—	—
Amaranthaceae	*Chenopodium glaucum*	0.09	—	—	—	—
Rubiaceae	*Galium spurium*	0.03	—	—	—	—
Boraginaceae	*Microula sikkimensis*	0.07	—	—	—	—
Liliaceae	*Allium sikkimense*	—	—	0.04	—	—
Polygonaceae	*Polygonum sibiricum*	0.05	—	—	—	—

Note: “—” indicates the absence of this plant at some stage. A: Short-term recovery patch; B: Long-term recovery patch; C: Healthy alpine meadow; D: Degraded alpine meadow; E: Bare Patch.

**Table 3:** Results of the KMO and Bartlett spherical tests.

Test Type		Test Results
Kaiser–Meyer–Olkin Measure of Sampling Adequacy		0.602
Bartlett’s Test of Sphericity	Approx. Chi-Square	575.368
	Df	6
	Sig.	0.000

**Table 4:** The portion of total variance explained.

Component	Initial Eigenvalues			Extraction Sums of Squared Loadings			Rotational Load Sum of Squares		
	Total	% of Variance	Cumulative %	Total	% of Variance	Cumulative %	Total	% of Variance	Cumulative %
1	3.895	97.378	97.378	3.895	97.378	97.378	2.152	53.811	53.811
2	0.100	2.505	99.883	0.100	2.505	99.883	1.843	46.072	99.883
3	0.004	0.100	99.983						
4	0.001	0.017	100.000						

**Table 5:** Matrix of the first two components after rotation.

Indicators	Principal Components	
	1	2
Species richnessX1	0.548	0.836
Shannon–Weiner Diversity IndexX2	0.714	0.699
Species evennessX3	0.844	0.536
Simpson Diversity IndexX4	0.794	0.607

**Table 6:** The coefficient matrix of factor scores.

Indicators	Principal Components	
	1	2
Species richnessX1	−1.463	1.955
Shannon–Weiner Diversity IndexX2	−0.014	0.394
Species evennessX3	1.390	−1.136
Simpson Diversity IndexX4	0.805	−0.496

**Table 7:** Scores of two factors at the five succession stages and the overall score.

Succession Stage	*F*1	*F*2	Composite Score
A	−11.272	16.768	1.655
B	−14.333	20.933	1.925
C	−16.318	23.774	2.164
D	−13.701	20.070	1.868
E	0.000	0.000	0.000

Notes: A: Short-term recovery patches; B: Long-term recovery patches; C: Healthy alpine meadow; D: Degraded alpine meadow; E: Bare Patches.

## Data Availability

The data presented in this study are available on request from the corresponding author. The data are not publicly available due to privacy.
